# ‘Trying to put a square peg into a round hole’: a qualitative study of healthcare professionals’ views of integrating complementary medicine into primary care for musculoskeletal and mental health comorbidity

**DOI:** 10.1186/s12906-018-2349-8

**Published:** 2018-10-29

**Authors:** Deborah Sharp, Ava Lorenc, Gene Feder, Paul Little, Sandra Hollinghurst, Stewart Mercer, Hugh MacPherson

**Affiliations:** 1 Centre for Academic Primary Care, School of Social and Community Medicine, University of Bristol, Canynge Hall, 39 Whatley Road, Bristol, BS8 2PS UK; 2 Primary Medical Care, Faculty of Medicine, University of Southampton, Aldermoor Close, Southampton, SO16 5ST UK; 3 General Practice and Primary Care, Institute for Health and Wellbeing, University of Glasgow, 1 Horseletthill Road, Glasgow, G12 9LX UK; 4 Department of Health Sciences, University of York, Heslington, York, YO10 5DD UK

**Keywords:** Primary care, Complementary medicine, Integrated medicine, Qualitative, NHS, Musculoskeletal, Mental health, Comorbidity

## Abstract

**Background:**

Comorbidity of musculoskeletal (MSK) and mental health (MH) problems is common but challenging to treat using conventional approaches. Integration of conventional with complementary approaches (CAM) might help address this challenge. Integration can aim to transform biomedicine into a new health paradigm or to selectively incorporate CAM in addition to conventional care. This study explored professionals’ experiences and views of CAM for comorbid patients and the potential for integration into UK primary care.

**Methods:**

We ran focus groups with GPs and CAM practitioners at three sites across England and focus groups and interviews with healthcare commissioners. Topics included experience of co-morbid MSK-MH and CAM/integration, evidence, knowledge and barriers to integration. Sampling was purposive. A framework analysis used frequency, specificity, intensity of data, and disconfirming evidence.

**Results:**

We recruited 36 CAM practitioners (4 focus groups), 20 GPs (3 focus groups) and 8 commissioners (1 focus group, 5 interviews).

GPs described challenges treating MSK-MH comorbidity and agreed CAM might have a role. Exercise- or self-care-based CAMs were most acceptable to GPs. CAM practitioners were generally pro-integration.

A prominent theme was different understandings of health between CAM and general practitioners, which was likely to impede integration. Another concern was that integration might fundamentally change the care provided by both professional groups. For CAM practitioners, NHS structural barriers were a major issue. For GPs, their lack of CAM knowledge and the pressures on general practice were barriers to integration, and some felt integrating CAM was beyond their capabilities. Facilitators of integration were evidence of effectiveness and cost effectiveness (particularly for CAM practitioners). Governance was the least important barrier for all groups.

There was little consensus on the ideal integration model, particularly in terms of financing. Commissioners suggested CAM could be part of social prescribing.

**Conclusions:**

CAM has the potential to help the NHS in treating the burden of MSK-MH comorbidity. Given the challenges of integration, selective incorporation using traditional referral from primary care to CAM may be the most feasible model. However, cost implications would need to be addressed, possibly through models such as social prescribing or an extension of integrated personal commissioning.

**Electronic supplementary material:**

The online version of this article (10.1186/s12906-018-2349-8) contains supplementary material, which is available to authorized users.

## Background

Mental health (MH) and musculoskeletal (MSK) conditions create a huge burden for patients, society and healthcare services. Globally, low back pain is the leading cause of disability [1], and in the UK MSKs account for 30% of GP consultations [2] and 30.8 million working days lost annually [3]. Mental ill health is the single largest cause of disability in the UK [4], uses more than 11% of the NHS (National Health Service) budget [5] and costs the UK economy £70–£100 billion/year [4]. Comorbidity of MH and MSK conditions is common - MH problems (anxiety or depression) are 4 times more common in those with persistent pain than in those without [6, 7] and MSK and MH conditions co-occur in 3% of working age (16–64 years) people in England [8]. People with low back pain are significantly more likely to have depression, anxiety and sleep disorders, and to be prescribed medication for these conditions, than those without [9]. Comorbidity is particularly concerning to GPs [10] and poorly addressed by current guidelines, evidence and practice [11], representing an ‘effectiveness gap’ (where available treatments are sub-optimally effective), which complementary and alternative medicine (CAM) may be able to fill [11–13]. CAM is commonly used by those with comorbid MH and MSK conditions [14, 15].

Although most commonly accessed privately in the UK, CAM can be integrated with conventional (NHS) care. Wiese and colleagues [16] describe three models of integration: 1) pluralism, a patient-based model, where the patient chooses which approach to use, in a ‘supermarket’ approach [17]; 2) selective incorporation, or integrated medicine, the co-optation of CAM by biomedicine, with CAM as an add-on, provided by trained conventional practitioners or CAM practitioners (on-site or off-site and funded by the NHS/patient/charity; and 3) integrative medicine or transformative integration, which aims to merge biomedicine into a new health paradigm incorporating a holistic approach and providing optimum treatment from any tradition [17–19]. This paper focusses on the second model. Compared to the consumerist approach of the first model, integrated and integrative medicine can promote continuity of care, address safety concerns, and reduce professional power struggles [20]. The third model, transformative integration, may still be a utopian ideal [19], whereas selective incorporation is preferred by biomedical staff [16]. In primary care, selectively incorporated CAM is more commonly delivered by CAM practitioners than conventional practitioners [21, 22]. Selective incorporation, where patients are referred from conventional healthcare to an off-site CAM practitioner, is similar to social prescribing, a system enabling primary care clinicians to refer patients to a broad range of community services, for example an exercise class or gardening club [23].

Many of the defining values of CAM are now considered part of mainstream care. These include patient-centred care and a holistic approach [24, 25], and emphasis on self-management and prevention, which are prominent goals in current UK health service policy planning [26, 27]. Person- and community-centred approaches to health and wellbeing have a key role in these plans, which can include CAM [28]. Primary care may be the area of the NHS where CAM would fit most comfortably, due to both primary care and CAM having a holistic outlook, emphasis on self-care and strong therapeutic relationships.

Primary healthcare professionals, including GPs, tend to be most positive about CAM for chronic self-limiting conditions or those with limited treatment options e.g. musculoskeletal [29] or chronic pain [29–31]. Other ‘effectiveness gaps’ include depression, anxiety and stress [13].There is very little research on CAM for comorbid MSK-MH. The sparse qualitative research with GPs and CAM practitioners about integration of CAM into publicly funded health care is rarely health condition-specific, and rarely addresses commissioning issues. Doctors’ views on CAM in general vary widely, from enthusiastic to sceptical, with sceptical or uncertain the dominant view [32], although one survey found that only 6% of primary care professionals were against integration of CAM [29]. Attitudes vary depending on the specific CAM approach – a survey of general practitioners (GPs) found that nearly 60% support acupuncture provision on the NHS [33]. Healthcare practitioners’ views on CAM are mainly based on professional rather than personal factors [34], in particular the limited evidence base [30, 32], although referral is often determined by patient preference [29, 35].

However, there are challenges to transformative integration and selective incorporation. Based on previous studies of generic integrative services, mainly from the point of view of conventional and CAM clinicians, these can include: preserving the epistemological stance of CAM, as conventional medicine tends to dominate [12, 19, 20]; differing ‘corporate cultures’ [36, 37]; professional conflicts; conventional practitioners’ lack of knowledge regarding CAM [38]; a lack of communication and collaboration between the two groups [37]; a limited evidence base for many CAM; and lack of time in NHS settings [31, 39]. Integration can also give rise to issues around regulation of quality and safety, and duty of care. This particularly applies to a referral model, given UK General Medical Council advice that GPs delegating care must be satisfied with the safety and quality of care, and the practitioner’s knowledge, skills and experience [40].

Integrated medicine may help to address comorbid MSK and MH conditions, but there is a lack of research specific to this clinical area. This study therefore sought to explore healthcare professionals’ views and experiences to identify the feasibility of integrating CAM for comorbid MH and MSK into UK National Health Service (NHS) primary care.

## Methods

We have followed COREQ guidelines in reporting this study [41].

This study explored the views and experiences of GPs, CAM practitioners and healthcare commissioners. This included their views of CAM and any experiences of CAM provision in an integrated fashion in NHS primary care settings; and their views on the potential for and challenges of integrating CAM into primary care, particularly for comorbid MSK and MH conditions.

For GPs and CAM practitioners, focus groups were conducted at three sites across England (A, B, C). A is a fairly large city in the south of England. B and C are moderately sized cities, B in the North and C in the South of England. For commissioners, a combination of focus groups and telephone interviews were conducted, as participants were located throughout England.

CAM practitioners were recruited through a variety of routes including the Complementary and Natural Healthcare Council (CNHC) mailing list and Facebook group, professional organisation online registers (CNHC, British Acupuncture Council, General Osteopathic Council, British Chiropractic Association, UK Tai chi union), Google searches, NHS hospital pain clinics using CAM, and NHS physiotherapy services. GPs were recruited by local CLRNs (Clinical Local Research Networks). Commissioners were recruited via an NHS management fellow at Bristol University, the project steering group, and commissioners of integrated medicine services in the UK. All potential participants were contacted by email, with telephone follow-up.

Sampling was purposive. For CAM practitioners, the criteria were type of CAM and NHS experience/training. For GPs they were practice location (urban/rural), practice socioeconomic characteristics, gender, ethnic background, attitudes to and experiences of CAM (as self-reported by potential participants in an email). We aimed to include commissioners with experience of commissioning CAM, particularly for MSK and MH, as well as in a variety of geographical locations. We did not collect data on reasons for non-response.

GP/CAM focus groups lasted 90 min and were held on university premises. Two researchers attended each focus group, one (AL) to lead the group and ask the questions, the other noting who spoke and non-verbal communication. AL is a senior research associate with experience of conducting interviews and focus groups, including a PhD using qualitative methods. Participants were offered payment for their time, for themselves or their employer. They were aware that the researcher was pro-CAM. The researcher aimed to maintain an objective stance regarding CAM during the interviews. Participants were assigned codes to ensure confidentiality. Topic guides were developed for the study (see Additional file 1). For CAM practitioners, questions focussed on experience in the NHS, experience treating patients with MSK and MH comorbidity, the evidence base for their therapy, relationships with GPs and barriers to integrating CAM into NHS primary care. GPs were asked about their experience of treating patients with comorbid MSK and MH, their knowledge and experience of CAM (in particular, referring their patients to CAM practitioners), and barriers to integrating CAM into NHS primary care.

Commissioners’ focus groups and interviews lasted between 15 and 60 min and were conducted by one researcher (AL). Interviews were either face–to-face, via telephone or video link. The choice between interview or focus group was based on participant preference and availability. Commissioners were offered payment for their time. The topic guide was developed for the study (see Additional file 1) and included questions about definitions and beliefs regarding CAM, experience of commissioning CAM, factors in commissioning decisions, experience of MSK and MH services, barriers to integration of CAM, and thoughts about what evidence might persuade them to commission a CAM service.

Digital audio recordings were transcribed verbatim by a professional company, with non-verbal communication added from our notes. Based on content analysis, a framework was used for all data analysis [42, 43]. Framework analysis is highly structured and systematic, providing a clear map of how analysis and interpretation were performed [42]. It facilitates constant reference back to the original data, to remain grounded [42], but is also structured around pre-set aims and objectives, allowing the answering of specific research questions in the participants’ language, in concordance with the abductive stance taken [44]. It consists of five key stages: familiarisation, identifying a framework, indexing, charting and mapping/interpreting [43]. The first four are mainly data management strategies, to order, sort, synthesise and condense the raw data, the bulk of interpretation takes place in the final mapping stage [43]. Data analysis was facilitated using Microsoft Excel and NVivo (computer assisted qualitative data analysis software developed to facilitate systematic and clear analysis) [45]. Familiarisation came through reading the transcripts. A framework of codes was developed from the data, with some a priori themes from the topic guides. Indexing involved comprehensively labelling all the data using the final framework, marking quotations (sentences, paragraphs) which belonged to a code. Charting was performed using the Framework function in NVivo, which uses a matrix, where each row was a participant and each column a code. A summary of the data was entered into each cell in the framework, using quotations as much as possible, with some synthesis and abstraction to make meaning clear [46] but using participant’s words and terms, to stay grounded in the data [42]. The final stage of mapping and interpreting was done in Microsoft Excel. Each column was interrogated for themes. At all stages the ‘strength’ of data was considered, which was based on the following criteria:

- Frequency (number of people) and extensiveness (length) of comments, not as absolute data but to provide an indication of importance [42].
- Specificity: quotes relating to a personal experience were considered more important than hypothetical references [47].
- Intensity or depth of feeling, for example, are the words positive, negative, middling [48]. Internal consistency (changes in individual’s views) was also considered [48]*.*
- Disconfirming evidence [49] and negative/deviant cases [50] either proposed alternative explanations, reinforced normative theories by providing unusual examples, explained individual variation from the norm, or refined theories

The study was approved by the University of Bristol Faculty of Medicine and Dentistry Research Ethics Committee (FREC) on 3rd July 2015, reference 21,603. Assurance was provided by the relevant NHS organisations for each of the sites.

## Results

Of the 55 CAM practitioners invited, 36 took part in 4 focus groups (65% response rate), two in Site A, one in Site B and one in Site C. Table 1 provides their details. Five practiced tai chi, four acupuncture, and three practiced each of yoga, mindfulness, hypnotherapy, osteopathy, massage. Two practiced nutritional therapy and two chiropractic, one practised homeopathy and one herbal medicine. Participants worked in a variety of settings: most were private but fourteen were located in the NHS, including GP practices, psychological therapy and pain clinics. Seven were NHS professionals (GP, consultant, nurse, occupational therapist, physiotherapist). Eleven were statutorily regulated (NHS professionals, osteopaths or chiropractors) and 21 voluntarily regulated (voluntarily registered with a regulatory body).

**Table 1:** Participants in CAM practitioner focus groups

Code^a^	CAM	Clinical setting	Statutorily regulated?	Voluntarily regulated?	NHS professional?	Practices in NHS?	Is your practice integrated into NHS?
A1.1	Mindfulness	Improving access to psychological therapies (IAPT), occupational therapy, pain clinic	YES	YES	YES	YES	YES
A1.2	Yoga	Private	NO	YES	NO	NO	NO
A1.3	Holistic massage, reiki	Private	NO	NO	NO	NO	NO
A1.4	Mindfulness	IAPT	NO	YES	NO	YES	YES
A1.5	Osteopathy	Private, in GP practice	YES	NO	NO	YES	NO
A1.6	Osteopathy	Private, in GP practice	YES	NO	NO	YES	NO
A1.7	Manipulation, Bach flowers, homeopathy, acupressure	General practice	YES	YES	YES	YES	YES
A1.8	Pilates, yoga	Private	Missing data				
A1.9	Massage, yoga (individual)	Private	NO	YES	NO	NO	NO
A2.1	Tai chi, qigong	Private; chronic patients	NO	NO	NO	NO	NO
A2.10	Homeopathy, Director of integrative medicine centre	Community interest company; NHS	NO	YES	YES	YES	YES
A2.2	Physiotherapy, adapted tai chi, Pilates	NHS rheumatology	YES	NO	YES	YES	YES
A2.3	Hypnotherapy	Private clinic with a physiotherapist	NO	YES	NO	NO	NO
A2.4	Massage, reiki	Private osteopathy clinic attached to a GP surgery	NO	YES	NO	YES	Yes
A2.5	Acupuncture	Low cost clinic	NO	YES	NO	NO	NO
A2.6	Acupuncture, meditation	Cancer centre, multi-bed clinic, community interest company	NO	YES	NO	NO	YES
A2.7	Tai chi	Private	NO	NO	NO	NO	NO
A2.8	Pain management	NHS pain clinic	YES	NO	YES	YES	YES
A2.9	Alexander technique, medical acupuncture	Nurse, NHS pain clinic	YES	YES	YES	YES	YES
B1	Tai chi	Private; collaboration with NHS	YES	YES	NO	YES (PREVIOUS)	SOMETIMES
B2	Mindfulness	Charitable; previously local educational authority	YES	NO	NO	NO	NO
B3	Mindfulness	Former GP; private	NO	YES	NO (retired GP)	NO	NO
B4	Microsystems Acupuncture	Private; charitable	NO	YES	NO	YES	NO
B5	Medical herbalist, nutritional therapist	Private	NO	YES	NO	NO	NO
B6	Tai chi	Primary and secondary care and community mental health	NO	NO	NO	NO	YES
B7	Yoga therapy	Private	NO	YES	NO	NO	NO
B8	Craniosacral, acupuncture, Kampo herbs	Private	NO	YES	NO	NO	NO
C1	Chiropractic	Private	YES	NO	NO	NO	NO
C2	Tai Chi and qigong	Private	NO	YES	NO	NO	NO
C3	Hypnotherapy	Private	Missing data				
C4	Chiropractic	Private	YES	NO	NO	NO	NO
C5	Yoga	Hospital; private	NO	YES	NO	NO	NO
C6	Physio	NHS Hospital	YES	N/A	YES	YES	YES
C7	Acupuncture, Chinese herbal medicine	Private	NO	YES	NO	NO	NO
C8	Hypnotherapy	Private; volunteer	NO	YES	NO	YES	NO
C9	Osteopathy, Heart Math, Alexander technique	Homeless health care; private	Missing data			YES	Missing data

^a^As two focus groups were conducted at Site A these are coded A1 and A2

Fifty-five GPs expressed an interest in participating, seven of whom subsequently declined and 28 could not attend due to timing. The final sample was predominantly based on GPs’ availability, although purposive sampling criteria were met. Twenty GPs (see Table 2) participated, in three focus groups, ten in Site A, six in Site B and four in Site C. Most stated their views as neutral or in favour of CAM, three were ‘sceptical’. Four practised CAM.

**Table 2:** Participants in GP focus groups

Code	Attitude to CAM^a^	CAM practitioner?	Deprivation in practice area (as reported by the GP)	Ethnicity	Practice location
A1	Neutral	No	Average	White	Semi-rural
A2	In favour	No	Deprived	Mixed race (Asian/Caucasian)	Urban
A3	Neutral but open	Yes, anthroposophic medicine	Mixed	Non-White	Urban
A4	In favour	Yes, acupuncture (British Medical Acupuncture Society, BMAS)	Deprived	White	Urban
A5	In favour	Previously (acupuncture, homeopathy)	Average	White	Semi-rural/ suburban
A6	Opposed to NHS funded CAM	No	Fairly deprived	White	Urban
A7	Mixed (depends on therapy)	Yes, acupuncture (BMAS)	Not deprived	White	Urban
A8	In favour	No	Not deprived	White	Semi urban
A9	Mixed (depends on therapy, payment etc)	No	Some deprivation	White	Urban
A10	In favour	No	Students	White	Urban
B1	Previously sceptical, becoming more open	No (acupuncture provided at surgery)	Deprived	White	Rural
B2	Neutral	No	Data missing	White	Locum
B3	Sceptical	No	Locum	White	Variety
B4	Open-minded but depends on the evidence	No (acupuncture provided at surgery)	Lower deprivation	Non-White	Suburban
B5	Data missing	No	Mixed	White	Data missing
B6	Data missing	Yes, acupuncture	Data missing	Data missing	Data missing
C1	Neutral	No	Affluent	White	Rural/urban
C2	In favour (if evidence-based)	No	Pockets of deprivation	White	Semi-rural
C4	Sceptical/neutral	No	Deprived	White	Urban
C5	Sceptical (but open to persuasion)	No	Mixed	White	Urban

^a^This is the respondent’s response to asking in an email “We are hoping that the focus groups comprise people with a diversity of opinion - would you say in general you are in favour of CAM, opposed to CAM or simply neutral?”

Of 30 commissioners invited, eight took part, most of whom were also GPs (Table 3). Six worked in CCGs (clinical commissioning group – NHS bodies responsible for commissioning local services), one in an integrated personal commissioning (a scheme using personal health budgets for patients/carers) demonstration site and one for the voluntary sector. One focus group was conducted with three participants; the others’ views were obtained through telephone interviews.

**Table 3:** Participants in commissioner focus groups/interviews

Code	Commissioning body/employer	Clinician?	Location in UK	Focus group or interview
1	CCG^a^	Former GP	South West	Focus group
2	CCG	GP	London	Telephone interview
3	CCG (pharmacy services)	GP	South West	Focus group
4	Integrated personal commissioning	No	South West	Telephone interview
5	CCG	GP	North	Telephone interview
6	CCG	GP	London	Focus group
7	CCG (self-care lead)	GP	South West	Telephone interview
8	Voluntary sector - social prescribing	No	North	Telephone interview

^a^Clinical commissioning group

The key themes arising from the data were: what is CAM; the role of CAM; feasibility of integrated medicine in the NHS; barriers to integration; GP education; regulation; and models of integration.

### What is CAM?

CAM was a difficult term for many GPs as it covers a wide range of interventions. Three GPs mentioned the ‘huge’ range of CAM and grouping this diverse range of treatments as ‘CAM’, was seen as ‘unhelpful’.

“I really, really struggle with this umbrella term of complementary and alternative medicine, because I see a huge spectrum” (GP A9)

Two described a spectrum of CAM based on effectiveness and safety, with chiropractic and osteopathy at one end and “mumbo jumbo”, e.g. homeopathy and reiki at the other. Some therapies – Pilates, yoga, tai chi, mindfulness and acupuncture – were not necessarily considered to be complementary, and exercise-based CAM – Pilates, tai chi, yoga – seemed to be more acceptable to GPs. Some were also more positive about CAM which ‘foster’ self-management.

“…nothing weird or wonderful there at all [acupuncture, tai chi, yoga], those are all things that are part of our everyday...I wouldn’t even particularly class any of those as complementary medicines” (GP A6)

“Self-care is so important. Teach someone to look after their sleep and not be so concerned about it, or to increase their core stability by using something for themselves, is much better than perhaps referring them to the homoeopathist and they lay out their store of symptoms again” (GP B5)

The most common criteria used to define CAM were its ‘philosophical approach’ and its lack of an evidence base. Six GPs talked about CAM as being treatments with a philosophy they perhaps did not accept or understand. For four GPs, the lack of evidence defined CAM, although another felt this did not distinguish it from conventional care. For commissioners, CAM was defined as treatment outside the mainstream.

“I suppose it’s [CAM] almost defined by what is in conventional, it’s the other things that are not considered conventional” (commissioner 7)

“I would say that anything that doesn’t have a solid evidence base would come under the principles of complementary medicine” (GP A6)

GPs discussed two particular areas of overlap between CAM and conventional medicine: exercise (e.g. tai chi) and social support (e.g. personal health budgets). For commissioners, CAM overlapped considerably with broader approaches such as social prescribing and holistic care.

### A role for CAM in primary care and MSK-MH comorbidity

All three groups felt that CAM had a role in the provision of primary care services, although GPs were the least enthusiastic and saw CAM’s role as limited. CAM practitioners were generally pro-integration.

Unsurprisingly, CAM practitioners were very positive about CAM, citing evidence for its effectiveness, and believed it to be commonly used and demanded by patients. The commissioners were generally positive about CAM, although this may reflect potential selection bias towards pro-CAM commissioners.

“I am very pro a more holistic approach” (commissioner 2)

GPs and CAM practitioners both saw MSK-MH comorbidity commonly in their practice. For GPs, common examples were fibromyalgia, “frequent attenders”/“heart sink patients”, overweight, back/chronic pain with anxiety/MH issues, and osteoarthritis. Many CAM practitioners gave examples of comorbidity and how CAM (in their opinion) could help treat it.

“I think most of the patients in general practice have more than one thing going on, so most patients with, you know, anxiety or depression have something else going on. Not all, but most, most I would say. Particularly perhaps when they get into their sort of 30s or 40s or whatever” (GP B2)

“there’s definitely an inter-connectedness, particularly with back pain and erm, mental health issues” (GP A9)

“I was just thinking I would love to see someone with just one problem. I was trying to think when was the last time? - I actually can’t remember” (CAM C6)

GPs and CAM practitioners both identified challenges in treating comorbidity, mainly NHS service issues, for example waiting lists for physiotherapy or pain clinics. CAM practitioners felt conventional treatment was often of limited benefit. Commissioners also recognised these challenges (although comorbidity per se did not tend to influence their decisions).

“I just feel that the services that we have to use on these people, such as the pain clinic and MATS [Musculoskeletal Assessment Triage Service] are often not meeting their needs” (GP A10)

“[Patients say] “Oh, well the GP just dishes out painkillers”, and it doesn’t solve the roots of their issue, their problem. So they’ll come to me. They say “I want a more holistic approach””(CAM A2.2)

There was some agreement across all three groups that CAM had a role in treating MSK-MH comorbidity, given the limited conventional treatment options or availability. Some GPs felt that something extra, possibly CAM, was needed to offer these patients. CAM practitioners explained that CAM can treat comorbidity using a holistic approach.

“those chronic pain patients who, we all know who they are in our practice, we all dread them popping up on our list, and we need something else to work with them, because more and more evidence says that actually up titrating opiates, has lots of implications, it isn’t good for our prescribing, it has lots of side effects for them. So we need something else to reach for, instead of our prescription pads, for these group of patients [chronic pain]. And I think that’s sort of the other side of it, that almost makes it a little bit exciting in the sense that it’s [integrative medicine] a new area that we could maybe tap into and get some real benefits” (GP B1)

### Is integrated medicine feasible in NHS primary care?

A number of GPs highlighted concerns that integrating CAM into NHS primary care would present challenges and might not be feasible. Although many of these concerns were only raised by a few GPs, the repeated emergence of the message across several themes justifies its inclusion as a key issue.

First, CAM was seen by a small number of GPs to be addressing much broader problems than those which primary care should be treating, described by two GPs as ‘first world problems’ – issues around wellbeing, preventative care, dis-ease. Similarly, some GPs saw CAM as a form of self-care overlapping with social support and exercise. This view of CAM contrasted with the GP’s primary role in treating disease.

“the extended, sort of, integration of integrated medicine is that there will be all of these services potentially who we could then refer into. And you’re creating the burden of dis-ease rather than disease, and then you’re increasing our burden” (GP A6)

Second, a small number of GPs, contemplating integrated medicine becoming part of their practice, thought it would involve fundamental changes to the GP consultation and communication i.e. becoming more patient-centred and ‘meaningful’. This was challenging, given the limitations and pressures of UK primary care (bureaucracy, overwork, time constraints).

“There’s lots of competing priorities though in terms of GP time, so where do you put complementary medicine as a priority?” (GP B4)

### Barriers to integration – The brick wall between CAM and NHS care

A central message, occurring across several themes (mostly from CAM practitioners), was the idea that CAM and conventional medicine have significant conceptual differences which are barriers to integration. The language used strengthens these data. CAM practitioners regarded CAM as holistic, promoting self-care and behavioural change, while conventional care was described as reductionist, paternalistic and passive. They perceived the conceptual differences between the “two worlds” of “mainstream medicine” and CAM as a barrier to integration.

“[CAM is] a completely different concept of really how the world is” (CAM A1.9)

“the Western approach is very much more reductionist, ooking for diagnosis. Whereas I think there’s a completely different approach from complementary therapies which is looking at a holistic and outward perspective. So there’s quite a lot of adjustments to be made which I think an NHS approach can’t cater for” (CAM C9)

Many CAM practitioners were concerned that attempts to overcome these differences would ‘secularise’, reduce and standardise CAM, and reduce the techniques practitioners could use, diminishing its value and holistic nature and reducing benefits. A few GPs concurred with this view, demonstrated by their concerns about feasibility of true integration in primary care.

“If you secularised qigong totally, if you strip it from all its, in a sense its spiritual value...if you take away the underlying principles in a sense, if you take away the theory and the philosophy… you leave it with a shell...just a form of exercise, a callisthenic, a dynamic movement exercise, a meditation without meditation” (CAM A2.1)

“there seems to be a sort of slight debate going on as to whether you could really, sort of, provide the range of services an osteopath would do privately within the NHS setting... a bit like trying to put a square peg into a round hole and whether or not you lose what, you know, what we think osteopathy is good for, or the good points” (CAM A1.6)

“I think the danger about being integrated into the Health Service if, if, if it stays as it is, is we’ll just be very limited as to what we can do” (CAM C4)

CAM practitioners saw CAM being used in the NHS more out of desperation - when conventional care fails or cannot offer anything more - than for its ability to prevent ill-health and promote wellness. They thought true and worthwhile integrative medicine would require a major change to conventional medical thinking, a view which some GPs also expressed. The only constructive suggestion for overcoming the gap between the ‘two worlds’ was through the planned changes in the NHS ‘Five Year Forward View (a policy document describing a new shared vision for the future of the NHS and new models of care which aimed to reduce health disparities and improve care).

For CAM practitioners, structural barriers such as NHS guidelines and bureaucracy were very challenging. Their emotional language emphasised the importance of this theme. Commissioners agreed that guidelines were very influential in their decisions. For GPs, key structural barriers were lack of time and competing priorities in GP consultations.

“…the therapists round here all have something to give, but at the moment we all just seem to be bashing our heads to a large extent against a large brick wall and hopefully this [project] is a chink in the wall” (CAM C8)

“[We] don’t have time during a GP consultation to give advice on CAM, you tend to move on to things which are more relevant to you as a GP, which you feel more confident about and which you have more knowledge about or can do something about” (GP B2)

Evidence of effectiveness appeared more important to CAM practitioners than GPs or commissioners. For CAM practitioners, evidence was the most important facilitator of integration and generating and implementing evidence was the biggest barrier.

“…that’s one of the things that’s incredibly difficult to get anything in to the NHS, it relies on evidence base. And, you know, whether it’s complementary or an orthodox approach, it’s got to have evidence base” (CAM C6)

For commissioners, the main factor influencing their commissioning decisions was evidence of cost-saving or affordability, and the current cold financial climate posed the biggest challenge to commissioning. Restrictive funding models were also seen as challenging, especially in general practice. CAM practitioners also recognised the importance of evidence of cost-saving which was ‘the only way’ to obtain NHS funding for CAM.

“…even drugs that come into us with really good evidence, um, we’re having to say, “where can you find the money to pay for this new treatment”” (commissioner 3)

“…everything has to be either cost neutral or saving money. That’s the kind of mantra, so it’s quite a difficult climate to suggest new services” (commissioner 7)

### GP knowledge

For GPs, a clear theme was the need to improve their knowledge and education about CAM, which commissioners and CAM practitioners agreed with. Lack of dialogue between the two professions was a related issue. The importance of GPs’ lack of knowledge and understanding of CAM reflects concerns that integration would extend the role of the GP beyond their current abilities or comfort zone.

“I would say my big barrier is my current understanding. I think it comes back to at the end of the day of my actual knowledge of what’s available and what’s proven erm, and locally what’s sort of available” (GP B1)

“…there’s a lack of education, formal education about complementary medicine at all, in GP training. We often just pick it up as we go along” (GP B4)

“So I think if you can even get [medical] students before they’re qualified to know what’s out there [CAM], know what the evidence base is, know who is regulated, know the training and the hoops that people have to jump through, I think it will be really helpful. I think the CCGs yes, but it’s too late, because you’ve got to get the GPs with that knowledge earlier” (CAM C6)

### Governance of CAM

Regulation of CAM practitioners was not a major issue for participants although some CAM practitioners felt that greater regulation of practitioners, and improved NHS awareness of regulation, were important. GPs did not mention regulation as a major factor, but that may be due to lack of awareness of the issues.

“I don’t see the chance of [hypnotherapy] getting integrated into NHS and NHS funded practice as long as there is a lack of regulation” (CAM A2.3)

“it’s giving confidence to the, to the GPs if they are referring to a CAM then if you are CNHC [Complementary and Natural Healthcare Council] registered, then there is a lot of, um, ground to that” (CAM C5)

Commissioners’ views varied on whether regulation of CAM practitioners would influence their decisions.

“…if it’s mainstream, those are fairly standard, for example, you know, a doctor or a nurse or a therapist for example, but when it comes to some of the alternative or complementary therapies then I don’t think always the systems are necessarily quite as rigorous” (commissioner 5)

“[Regulation] is something really that I do not want...imposed on all these other people [CAM practitioners]...The regulation in the health service is an unmitigated disaster now and is costing the system a fortune with...no evidence that it improves quality” (commissioner 6)

### Models of integration

CAM practitioners, GPs and commissioners all felt that CAM might address some limitations of NHS provision for patients with MSK-MH comorbidity. For example, where waiting times for NHS treatment were long or the course of treatment/consultations too short; where lifestyle change or an active approach could reduce secondary care burden; where additional treatment options were needed; or to create a more holistic service.

“People, at the moment, are frustrated because they’re, they’re going to doctors and they’re being like, sometimes given just an option of pain relief or physio, but there’s a waiting list which is too long for them” (CAM A2.2)

CAM practitioners varied in their views as to whether paying for CAM can improve commitment, adherence, and its perceived value, and that co-payment by patients, on a sliding scale depending on ability to pay, might be the best model. This was also seen as a way of raising awareness of the cost of healthcare, including NHS care, which is often not clear to patients.

“I would see that you would have perhaps council paying a third, NHS paying a third, and it would be wonderful if the patient paid a third to show a commitment. Would be a nice vision. Would help with the cost saving [laughs].” (CAM A1.2)

Commissioners suggested models for integrating CAM into NHS services. The most promising appeared to be integrated personal commissioning budgets (a scheme using personal health budgets for patients/carers to take more control over their health, and to integrate health, social care and voluntary services) and social prescribing, although the available data have limited generalisability and these models are wider than just CAM. Signposting to CAM (mentioning it without formally referring patients) was also mentioned. Alternatives to NHS-funding were suggested, including charity-funding, voluntary practitioners and public-sector funding. Other considerations included improving communication between CAM and NHS practitioners (which was reported as poor by GPs), and providing CAM through a social enterprise.

## Discussion

### Summary of findings

GPs, CAM practitioners and commissioners agreed that CAM may be useful to address the limitations of NHS care for the prevalent issue of MSK-MH comorbidity, which include availability and limited effective treatments. Exercise- or self-care-based CAMs were the most acceptable to GPs.

Although they agreed that MSK-MH comorbidity is prevalent and burdensome and needs a new approach, the three groups’ views on the barriers to using CAM within the NHS varied. A central message regarding integration was the different understandings of health between CAM and conventional medicine, which were likely to impede integration. CAM practitioners and GPs were concerned about integration fundamentally changing the care they provide, and both groups agreed that GPs’ lack of education, knowledge, and understanding regarding CAM was a barrier to integration. For CAM practitioners, NHS structural barriers were a major hurdle. For GPs, lack of time and resources and current pressures were important issues, causing them to feel integration of CAM was beyond their capability. GPs emphasised that integrated medicine would have to relieve their burden rather than add to it. In terms of facilitating integration, evidence was more important to CAM practitioners than GPs and certainly than commissioners, who were more focussed on cost saving. Governance was not a major issue.

Various models of integration were discussed, with little consensus. GPs and commissioners saw an overlap of CAM with social support and exercise and current UK policy regarding self-care and patient activation. Integration could therefore be seen as one facet of social prescribing and holistic GP care.

### Comparison with previous literature

A systematic review has confirmed that GPs see comorbidity as challenging to treat [51]. Our results support previous findings that GPs see MSK pain as an effectiveness gap suitable for an integrated/integrative approach [12, 13, 29, 30], and suggest this also applies to MSK-MH comorbidity. GPs’ preference for exercise- or self-care- based CAM aligns with UK healthcare guidelines for low back pain (NICE guideline NG59), depression (NICE guideline CG91) and anxiety (NICE guideline CG113).

Our findings confirm previously identified challenges of integration that are recognised by UK healthcare professionals and may apply to MSK-MH comorbidity. These include: different ‘world-views’ in understanding health/health care [16, 37, 52]; concerns about secularising CAM when integrating [12, 19, 20] or having to fundamentally change conventional care [16]; NHS bureaucracy (for CAM practitioners) [31, 53]; GPs’ lack of knowledge and need for education in CAM [54–56]; and lack of time in NHS settings [31, 39]. GPs’ concern that integration of CAM was beyond their current capacity appears to be a new finding and is discussed under Implications below. Although we focussed on an integrated (selective incorporation) model in our topic guides, the challenges raised by participants, particularly those regarding the conceptual differences between CAM and biomedicine, are more pertinent to a transformative model of integration –described by GP A6 as “the extended…integration of integrated medicine”. They confirm the view that transformative integration may be a ‘utopian ideal’ [19].

The concern about ‘trying to put a square peg into a round hole’ - the ‘secularisation’ of CAM - is raised by Hollenberg & Muzzin, as ‘colonisation’ of CAM [57]. Wiese and colleagues found that incompatibility between the ethos of science and CAM mean integration often involves ‘co-optation’ of CAM, and biomedical domination. There are examples of such secularisation in mindfulness-based approaches and herbal medicine [58, 59].

Poor GP knowledge implies education is needed about CAM – in the UK GPs are keen [29] and in the USA, CAM is often part of the medical curriculum [60]. Inter-professional education is an option [61].

The relatively low importance commissioners gave to evidence is interesting, but confirms findings from conventional medicine [62]. That CAM practitioners believe evidence is important has been reported before [63, 64]. However, CAM practitioners may lack research training [65], and have concerns about the appropriateness of traditional research methodology in CAM [66, 67]. Commissioners’ emphasis on cost-saving evidence reflects an emphasis on prioritisation of health service funding [68] and more economic evidence is needed for CAM [69].

### Implications

In our study, all three groups of healthcare professionals believed that an integrated approach using certain CAM may be worth pursuing to address limitations of conventional approaches in treating MSK-MH comorbidity, but they had different concerns about how an integrated approach might be implemented.

Findings highlight the burden that GPs are carrying in the UK – their workload has substantially increased [70, 71], a significant proportion of which is MSK and MH conditions [72, 73]. This ‘crisis’ creates reluctance to even contemplate anything new, e.g. integrated medicine, even if potentially beneficial. GPs and commissioners both felt successful integrated medicine would need to relieve NHS pressures, by reducing GP burden and costs. Integrating CAM may relieve GP workload for patients with limited treatment options [37]. Our study confirms 2003 findings that GPs and commissioners see integration of CAM as potentially helping to meet NHS targets [68]. Current policy drivers include the self-care and patient activation components of the NHS England Five Year Forward View [25, 28], in which primary care is central [26]. This aligns with “expansionism”- which favours the inclusion of alternative approaches [26, 74] e.g. social prescribing and holistic care. Conversely, some GPs’ concerns about integration reflect “reductionists’” arguments for GPs to reduce their duties to focus on the “genuinely vulnerable and sick” [75]. This is in line with the 2004 General Practitioner contract which has resulted in GPs practicing a more biomedical model of health and illness [76].

In terms of an integration model, transformative models are unlikely to be successful due to severe restrictions on NHS spending and concerns that these models would necessitate secularisation of CAM or fundamentally changing conventional care [77]. Instead, selective incorporation using referral from NHS primary care, as in social prescribing, may help the NHS address the needs of comorbid patients. Social prescribing is increasingly popular, with a national social prescribing network [78], and funding for social prescribing schemes/interventions from the UK Department of Health [79]. Regulatory implications - GPs would need to be sure of CAM practitioners’ regulation, quality and safety - may necessitate CAM practitioners becoming allied health practitioners, facilitated by the Professional Standards Authority’s CAM registers [80]. This referral model would require GP education and referral protocols/guidelines [20, 56], and has cost implications, as CAM is almost always patient-funded or part-funded [81, 82]. Co-payment by patients/NHS may be an option, but has equity implications and would need to consider ability to pay, particularly as MSK-MH comorbid patients tend to be of lower socioeconomic status [83, 84]. The King’s Fund recently rejected the controversial issue of patients paying for NHS treatment [85]. Another funding option is public health funding, given the overlap between integrative medicine, preventative medicine and public health [86].

For anyone attempting to integrate CAM into a conventional health system we suggest: identifying the evidence for effectiveness and cost-effectiveness; careful consideration of terminology; working with practitioners to develop a CAM approach which respects the philosophies of both conventional medicine and CAM; considering exercise- or self-care-based CAM; including education for GPs; and linking to relevant conventional health policies/strategic priorities e.g. in the UK the Five Year Forward View [56].

There is a need for more evidence of effectiveness and particularly cost-effectiveness of CAM; MSK-MH comorbidity is a fertile area for research. Exercise- and self-care-based CAM may be the best approaches to evaluate as they appear to be most acceptable to GPs.

### Strengths and limitations

We were successful in recruiting a large number of practitioners, however we did not aim for data saturation so a larger sample may provide new themes or understandings. Purposive sampling captured the views of a wide range of individuals, and we met all the criteria in our sampling frame, despite GPs’ limited availability. However, the professionals who took part were likely to have a more pro-CAM stance than average, which may mean our results are skewed towards the positive aspects of an integrated approach. The researcher’s pro-CAM stance may have biased responses although we made efforts to emphasise that we were interested in a range of views and remaining grounded in the objective data from the literature review phase. Commissioners were very difficult to recruit, due to lack of a central organising body or mailing list, and busy schedules. For the large part, we relied on personal contacts, giving a skewed sample with mainly positive experiences regarding commissioning CAM. Their limited availability to attend a focus group necessitated more one-on-one interviews, which may have influenced the findings. More research with commissioners would be very valuable.

## Conclusions

GPs, commissioners, and CAM practitioners felt that integration of CAM may offer a useful solution to the challenges faced by the NHS in treating MSK-MH comorbid patients. However, integration of CAM into NHS care/settings for these patients is limited by structural barriers, philosophical differences and concerns about changing both types of care fundamentally. Selective incorporation using referral from NHS primary care into CAM services may be a feasible model of integration, although cost implications would need to be addressed, possibly through models such as social prescribing or co-payment. Regulatory issues would also need to be addressed, including raising GPs’ awareness of CAM registers.

## Additional file

Additional file 1: Focus group/interview topic guides. (DOCX 28 kb)

## Abbreviations

CAM: Complementary and alternative medicine
CCG: Clinical commissioning group
MH: Mental health
MSK: Musculoskeletal
NHS: National Health Service
RCT: Randomised controlled trial
