# Supplementary material for: ‘Trying to put a square peg into a round hole’: a qualitative study of healthcare professionals’ views of integrating complementary medicine into primary care for musculoskeletal and mental health comorbidity
Source: BMC Complement Altern Med. 2018 Oct 29;18:290. doi: 10.1186/s12906-018-2349-8 (PMC6206651; doi:10.1186/s12906-018-2349-8)
Supplement: Supplementary file 1 — Focus group/interview topic guides. (DOCX 28 kb) [file 12906_2018_2349_MOESM1_ESM.docx]

**FOCUS GROUP TOPIC GUIDE CAM Practitioners** *(v4.0 Nov 2015)*

**Preliminaries**

- Welcome and introductions
- Explain how the focus group will work – pseudonyms, recording, analysis, confidentiality, note-taking.
- Give a project overview including aims and structure, emphasising we are interested in primary care, adults and certain types of MSK and MH. Our definition of CAM/IM (list included therapies & practitioner-only)

**Questions and prompts:**

1. Very briefly please could you each tell the group what CAM therapy you practice, which setting/s you work in (including if it is NHS), registration, and roughly how long you have been practicing?
2. From your experience, what are the differences between NHS and private practice (e.g. benefits, challenges, patients, logistics)? For those of you who don’t work in the NHS is it something you would like to do? Why/why not?
3. What does ‘integrated’ practice mean to you? Do you think your practice is integrated?
4. Do you think CAM should be integrated into primary care (general practice)? Why/why not? How? Are there any CAMs you think are more or less likely to be successfully integrated? Why?
5. What do you think is important to GPs (and other NHS clinicians) when considering to refer to/recommend CAM practitioners e.g. qualifications/experience/registration Prompt: What relationship and dialogue do you have with GPs and other primary care staff?
6. What do you think are the barriers to the integration of CAM for patients into UK general practice? Particularly for musculoskeletal and mental health.

Prompts:

- 1. Commissioning/money
  2. Patient/public attitudes
  3. NHS staff attitudes
  4. Availability of providers
  5. Evidence
  6. Different philosophies (CAM/conventional)
  7. Any local barriers?

1. Please could you tell me a bit about your experience of treating patients with multimorbidity (musculoskeletal and mental health issues)? Prompt: How do you address patients’ mental/emotional state?
2. Are you aware of the evidence for your therapeutic approach?
3. In our literature review we found that the evidence gaps (where there is some evidence for ‘proof of concept’ but definitive research is still needed) are

Mindfulness/meditation/MBSR – Stress, depression, anxiety, MH in chronic disease (also sleep for meditative movement)

Acupuncture – FMS, neck pain, sleep, depression

Tai chi – OA, anxiety

Yoga – LBP, depression

Would you agree? Why/why not? What other gaps do you think exist? Do you think these ideas could be researched in, say, an RCT?

1. In our project so far we found that some of the common models of delivery for IM are

- CAM practitioners working privately within NHS settings e.g. GP surgeries, either with or without GP referral
- NHS clinicians e.g. GPs or physios, (or other providers e.g. exercise professionals) providing the CAM themselves
- Private CAM services being contracted to provide CAM for NHS patients
- Charity funded (or reduced fee) CAM provided to NHS patients

Do you have experience of any of these models? Can you see any drawback or benefits from your perspective? Are there any other models you are aware of?

1. In our literature review we found that some of the common outcomes measured in trials of IM for MSK/MH are pain, function, disability, quality of life, wellbeing, general health, anxiety, depression, fatigue, sleep, stress, mood, cost effectiveness, adverse events. Do you think these are the right outcomes**?** Do you measure outcomes yourself? What other outcomes would you suggest? What would be your number one/primary outcome?
2. Taking into account our literature review findings and the discussion we have just had, what do you think we should focus on studying in a trial in this area? Prompts: therapies, patient population (conditions, demographics), model of delivery, outcome measures, commissioning options/funding arrangements.
3. Any other comments or suggestions?
4. Finally, are you aware of anywhere where CAM is being provided on the NHS or by NHS professionals or referral, which you think we should have included and/or we should consider including for our case study phase?

**FOCUS GROUP TOPIC GUIDE GPs** *(v4.0 Nov 2015)*

**Preliminaries**

- Welcome and introductions
- Explain how the focus group will work – pseudonyms, recording, analysis, confidentiality, note-taking.
- Give a project overview including aims and structure emphasising we are interested in integrated medicine (define), primary care, adults and certain types of MSK and MH

**Questions and prompts:**

1. Please could you tell me a bit about your experience of treating patients with multimorbidity particularly those with mental health and musculoskeletal issues?
2. What therapies would you define as CAM (prompt re manipulation /osteo /chiro/physio mindfulness/tai chi/pilates/yoga)? What knowledge do you have of CAM and of its benefits/dis-benefits? Do you believe that CAM works and if so how? Do you know what CAM is on offer on the NHS and how to access it? Where do you get that information?
3. Please could you tell me a bit about your experience of complementary medicine in your practice? e.g. signposting to CAM; referring to CAM; recommending CAM; discussing CAM use with patients
   1. How do you choose which CAM therapies and/or practitioners? Is avoiding medication a factor?
   2. How do you decide which conditions and/or patients are suitable for CAM? – prompts: if a patient asks, as a last resort, acute vs chronic pain, MSDs, MH
4. What are the local pathways available for your patients to access CAM?
5. Are you aware of any barriers/facilitators to CAM use for your patients? If yes, what do you think they are?
6. What do you think are the **barriers and facilitators** to the integration of CAM for patients in UK general practice? Prompt: what would you need in order to provide access to CAM (on the NHS) for your patients?
   1. Funding/commissioning/money
   2. Patient/public attitudes
   3. GP/NHS professionals attitudes
   4. Availability of providers
   5. Regulation of CAM practitioners
   6. Evidence
   7. GP fear of reprisals
7. In our literature review we found that the evidence gaps (where there is some evidence for ‘proof of concept’ but definitive research is still needed) are

Mindfulness/meditation/MBSR – Stress, depression, anxiety, MH in chronic disease (also sleep for meditative movement)

Acupuncture – FMS, neck pain, sleep, depression

Tai chi – OA, anxiety

Yoga – LBP, depression

FMS and CFS – may be challenging?

Would you agree? Why/why not? What other gaps do you think exist? Do you think these ideas could be researched in an RCT?

1. In our project so far we found that some of the common models of delivery for IM are

- CAM practitioners working privately within NHS settings e.g. GP surgeries
- NHS clinicians e.g. GPs or physios providing the CAM themselves Training NHS or exercise staff (exercise on referral) to deliver CAM e.g. teach tai chi
- Private CAM services being contracted to provide CAM for NHS patients

Do you have experience of any of these models? Do you think they would work? Are there any other models you are aware of?

1. In our project so far we found that some of the common models of funding IM are

- NHS funded - CAM staff are NHS staff
- NHS funded – private/CiC CAM services commissioned by NHS
- Part funded by NHS/charity/other organisation (patient pays a reduced fee)
- Paid for by health insurance

Do you have experience of any of these models? Do you think they would work? Are there any other models you are aware of?

1. In our literature review we found that the some of the common outcomes measured in trials of IM for MSK/MH are pain, function, disability, quality of life, wellbeing, general health, anxiety, depression, fatigue, sleep, stress, mood, cost effectiveness, adverse events. Do you think these are the right outcomes? What other outcomes would you suggest?
2. Do you think cost effectiveness is important?
3. Taking into account our literature review findings and the discussion we have just had, what do you think we should focus on studying in a trial in this area? Prompts: therapies, patient population (conditions, demographics), model of delivery, outcome measures, commissioning options/funding arrangements.
4. Any other comments or suggestions?

**FOCUS GROUP TOPIC GUIDE** *(v4.0 Feb 2016)* ***Commissioners***

**Preliminaries**

- Welcome and introductions
- Explain how the focus group will work – pseudonyms, recording, analysis, confidentiality, note-taking
- Give a project overview including aims and structure, what is CAM and IM (integrative medicine)

**Questions and prompts:**

1. What approaches would you define as Complementary and Alternative Medicine? (prompt re manipulation/osteo/chiro/physio/mindfulness/tai chi)?
2. Have you ever commissioned or considered commissioning or decided not to commission a CAM service? What was the service and what was it for? Why did you commission it? Can you give me a specific worked example?

Are you aware of any local services which are integrating CAM with mainstream /conventional medicine or provide a potential opportunity for CAM integration?

1. Do you believe that CAM works and how? Does this differ when you are in your commissioner role compared to you personally?
2. How do you make your commissioning decisions? How structured is your decision-making? [leave as open question but check for the following:]
   1. Evidence - what is good evidence for you? where do you look?
   2. Guidelines
   3. Cost – cost effectiveness, opportunity cost, savings, QIPP
   4. Patient benefit - long term benefits/prevention/self-management
   5. Patient demand
   6. Wider benefits e.g. mental health or wellbeing/ multi – morbidity?
   7. Safety
   8. Views of authority e.g. for accountability
   9. Regulatory status of the practitioners/profession
   10. Political influences
3. Is the commissioning process different for CAM?
4. Do patient pressure groups have any influence on your decisions? Is CAM a contentious issue for you and if so, how does this compare to other interventions/conditions e.g. cancer drugs?
5. Do you have essential criteria for the practitioners in your services e.g. regulation, training etc? How do you set standards for the services you commission? Particularly thinking of CAM practitioners and their regulation etc
6. Please could you tell me a bit about your experience and knowledge of commissioning for musculoskeletal and mental health issues?
7. Do you take multi-morbidity into account when you commission? Especially for musculoskeletal and mental health. If yes, how? If not, do you think you should?
8. What do you think are the barriers to the implementation of IM for patients with MSD/MH in UK general practice?

Prompts:

- 1. Commissioning/money/funding
  2. Patients’ attitudes
  3. NHS professionals’ attitudes
  4. Availability of providers
  5. Evidence

1. What might persuade you to commission an IM service? In our literature review we found that the evidence gaps (where there is some evidence for ‘proof of concept’ but definitive research is still needed) are

Mindfulness/meditation/MBSR – stress, depression, anxiety, mental health problems in chronic disease (also sleep for meditative movement)

Acupuncture – Fibromyalgia, neck pain, sleep, depression

Tai chi – osteoarthritis, anxiety

Yoga – low back pain, depression

Would you agree? Why/why not? What other gaps do you think exist?

1. In our project so far we found that some of the common *models of delivery* for IM are

- CAM practitioners working privately within NHS settings e.g. GP surgeries, either with or without GP referral
- NHS clinicians e.g. GPs or physios providing the CAM themselves
- Private CAM services being contracted to provide CAM for NHS patients
- Charity funded (or reduced fee) CAM provided to NHS patients
- Training NHS or exercise staff to deliver CAM e.g. teach tai chi
- Payment through insurance

What do you think about these models? Do you have experience of any of these models? Do you think they would work? Are there any other models you are aware of?

1. In our project so far we found that some of the common *models of funding* IM are

- NHS funded – (CAM staff are NHS staff)
- NHS funded – private/CiC CAM services commissioned by NHS
- Part funded by NHS/charity/other organisation (patient pays a reduced fee)
- Paid for by health insurance
- Personal health budgets/ integrated personal commissioning (not found any but has been mentioned in focus groups)

What do you think about these models? Do you have experience of any of these models? Do you think they would work? Are there any other models you are aware of?

1. Given that some studies have shown that commissioners’ decisions aren’t always based on trial evidence, what kind of trial might persuade you to consider commissioning an IM service - therapies, patient population (conditions, demographics), model of delivery, outcome measures, commissioning options/funding arrangements.
2. In particular, in our future trial, what outcomes would persuade you to consider commissioning our IM service? Would these be the same outcomes you would expect to measure in a service evaluation? In our literature review we found that some of the outcomes measured in trials of IM are pain, function, disability, quality of life, wellbeing, general health, anxiety, depression, fatigue, sleep, stress, mood, cost effectiveness, adverse events.
3. Any other comments or suggestions?
